# Virtual Chromoendoscopy in Capsule Endoscopy: A Narrative Review

**DOI:** 10.3390/diagnostics12081818

**Published:** 2022-07-28

**Authors:** Alexandros Toskas, Faidon-Marios Laskaratos, Sergio Coda

**Affiliations:** 1 Wolfson Unit for Endoscopy, St Mark’s Hospital, Harrow HA1 3UJ, UK; flaskaratos@nhs.net; 2 Digestive Diseases Centre, Barking Havering and Redbridge University Hospitals NHS Trust 2, Dagenham RM7 0AG, UK; sergio.coda@nhs.net; 3 Photonics Group, Department of Physics, Imperial College London, London SW7 2AZ, UK

**Keywords:** capsule endoscopy, chromoendoscopy, FICE

## Abstract

The usefulness of virtual chromoendoscopy (VC) in capsule endoscopy (CE) isa controversial issue, with conflicting studies regarding its efficacy. FICE and a blue filter were embedded in the PillCam^TM^ software, with the aim to assist readers in identifying the source of obscure gastrointestinal (GI) bleeding (OGIB), coeliac disease mucosal changes and other small and large bowel lesions, including polyps and tumors. This review aims to summarize the existing evidence on the value of VC in the visualization and identification of different types of pathology. Overall, VC in CE with FICE 1 and 2 can be a useful adjunctive tool and may increase the visibility of pigmented lesions, such as angiectasias and ulcers. However, it does not appear to improve the detection of polyps or tumors. On the other hand, the role of FICE 3 and the blue filter appears to be limited. FICE may also be helpful in differentiating hyperplastic and adenomatous colonic polyps during colon capsule endoscopy, although more evidence is needed.

## 1. Introduction

Video capsule endoscopy (VCE) is a minimally invasive endoscopic modality, which was initially introduced for the investigation of the small intestine, but currently a range of capsules are available that can facilitate the inspection of the entire GI tract [1]. The role of virtual chromoendoscopy (VC) in capsule endoscopy has been investigated in previous studies, but has not gained wide acceptance in clinical practice, unlike the use of VC in conventional fibreoptic endoscopy [2]. Several types of VC are integrated in modern endoscopes, enabling the inspection of microvascular and surface patterns, such as narrow band imaging (NBI; Olympus, Tokyo, Japan), Fuji intelligent color enhancement (FICE; Fujinon Inc., Saitama, Japan), and I-Scan (Pentax, Tokyo, Japan). Recently, FICE technology and the blue mode have been included into the RAPID software of VCE [2]. 

Flexible spectral imaging color enhancement (FICE) is a form of virtual chromoendoscopy that is incorporated in the capsule reading software and can be used by reviewers to enhance the delineation of lesions in the small bowel [2]. FICE technology decomposes images by using specific wavelengths (red, green, and blue) and then directly recreates the images with enhanced surface contrast. This leads to enhancement of tissue microvasculature, because of the different optical absorption of light by hemoglobin in the mucosa. On the other hand, blue mode imaging shifts the color within a short wavelength range of 490 to 430 nm, superimposed on the regular white light. Both of these technologies provide real-time enhancement of the surface patterns and color gradients of the GI mucosa, with the intention to better depict small differences between adjacent mucosal areas [3].The FICE wavelength settings are as follows: FICE 1 (red, 595 nm; green, 540 nm; blue, 535 nm); FICE 2 (red, 420 nm; green, 520 nm; blue 530 nm); and FICE 3 (red, 595 nm; green, 570 nm; blue, 415 nm). FICE 1 reduces the bile interference, FICE 2 emphasizes blood, and FICE 3 emphasizes the differences between bile and blood [4]. However, despite the presumed theoretical advantages, data on the application of virtual chromoendoscopy in VCE are limited [5] and the ideal settings for better recognition of the various lesions that can be found in the small bowel and the remaining GI tract are not studied adequately [3]. This review aims to summarize the existing data regarding the use of different modalities of virtual chromoendoscopy.

## 2. Materials and Methods

**Figure 1:**
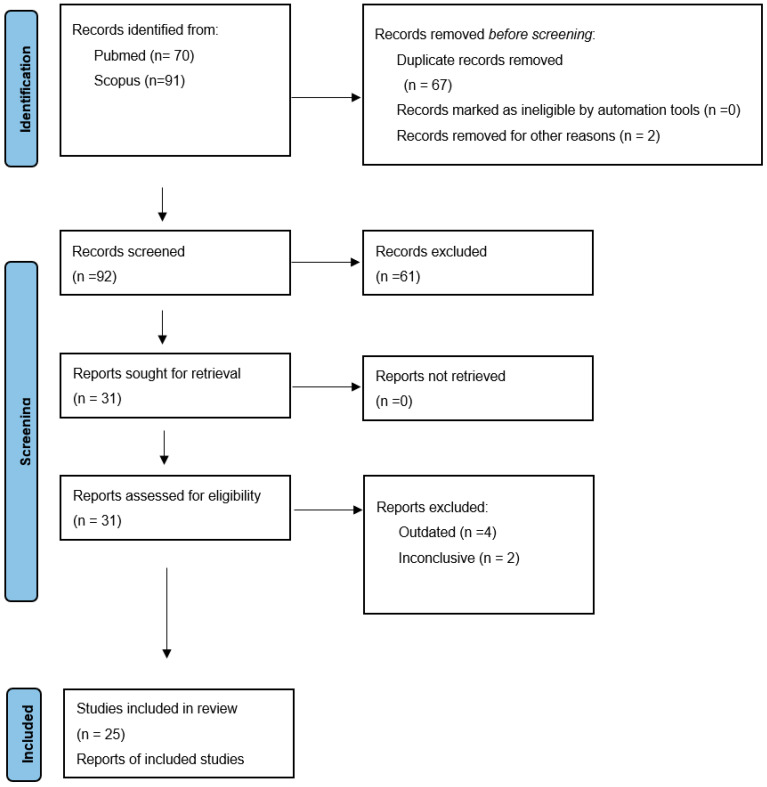
PRISMA flow diagram.

Using the PRISMA guidelines, 2 databases were searched (Pubmed and Scopus), using the keywords “Capsule”, “Chromoendoscopy”, “FICE”. The results were filtered for those available within the last decade when FICE was integrated in capsule software. In total, 169 results were found. Duplicates or the irrelevant publications to our subject were removed. Finally, 25 reports were studied in full text (Figure 1).

## 3. Discussion

The role of virtual chromoendoscopy has been investigated mainly in small bowel capsule endoscopy and more recently, in colon capsule endoscopy also.

### 3.1. Small Bowel Capsule Endoscopy

#### 3.1.1. Angiectasias, Erosions/Ulcers and Tumors

**Figure 2:**
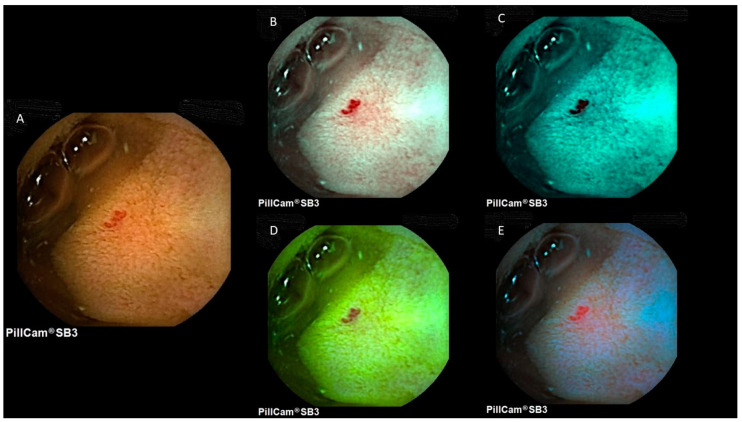
Image of a small bowel angiectasia at PillCam SB3. (**A**) White light; (**B**) FICE1; (**C**) FICE2; (**D**) FICE3; (**E**) blue mode.

**Figure 3:**
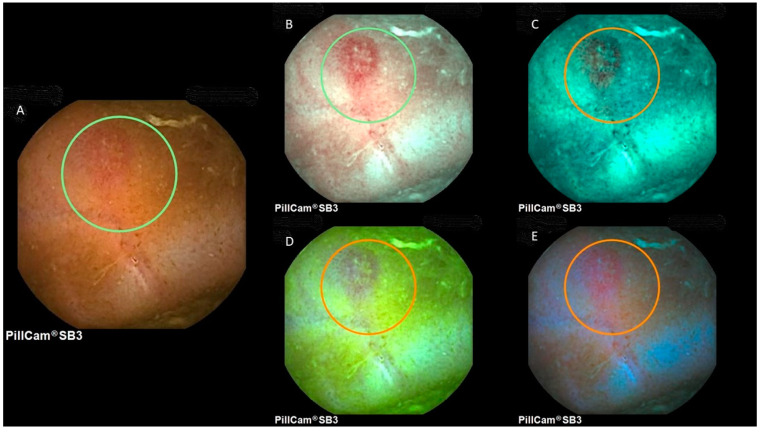
Image of a small bowel aphthous ulcer at PillCam SB3. (**A**) White light; (**B**) FICE1; (**C**) FICE2; (**D**) FICE3; (**E**) blue mode.

The detection of small bowel lesions using virtual chromoendoscopy settings in VCE is a controversial issue, with studies reporting contrasting results [1,4,6]. Figure 2 and Figure 3 provide representative images of a small bowel angiectasia and aphthous ulceration, respectively, using white light, FICE1, FICE2, FICE3 and the blue mode.

Ogata et al. in their cohort of 24 patients found a significantly increased visibility and detectability of small bowel angioectasias, ulcers and erosions with the use of contrast imaging (e.g., FICE) and BF versus standard mode CE [7]. Da Silva et al., in a single center retrospective study of 22 patients, assessed the benefits of chromoendoscopy in the Mirocam^TM^ system in 100 pictures from small bowel lesions, including angioectasias, ulcers and erosions. Two independent gastroenterologists assessed those images. For each of the different modalities, there was no significant difference between virtual chromoendoscopy and normal white-light endoscopy (*p* < 0.001) [2]. Rimbas et al., in a retrospective single center study, selected 250 difficult-to-interpret small-bowel ulcerative and 50 artifact lesions selected from 64 VCE recordings. These were reviewed by four experienced VCE readers initially with white light imaging (WLI), then with the addition of all available virtual chromoendoscopy presets (FICE 1, 2, and 3 and blue filter). Overall, this study showed that chromoendoscopy increased the detectability of ulceration compared to WLI (*p* < 0.05). FICE 1 and 2 were found to be useful but blue filter and FICE 3 were found to be misleading [8]. Nakamura et al., in a comparative study of 50 patients with angiodysplasia, compared the sensitivity and specificity in the detection of angiodysplasia between conventional CE and FICE. Two experienced doctors reviewed the images. The FICE reading had statistically better sensitivity (91% vs. 80%) (*p* < 0.01) but less specificity (86% vs. 100%) [3]. In the study by Sakai et al., 4 inexperienced gastroenterologists reviewed 12 VCE with the aim of measuring the detectability of small bowel lesions using FICE. FICE settings 1 and 2 significantly improved the detectability of angioectasia (*p* = 0.0017 and *p* = 0.014, respectively) and erosions/ulcerations (*p*  =  0.0012 and *p*  =  0.0094, respectively) [9]. In another study by Cotter et al., 49 VCEs were reviewed and the visibility of angioectasias, ulcers/erosions and villous edema/atrophy detected by CE improved significantly with the use of FICE-1 and FICE-2. Overall, the delineation of lesions was improved in 77% of cases with FICE 1, 74% with FICE 2, 41% with FICE 3 and 39% with the BF [10]. Similarly, in the study by Sato et al., SBCE images from 189 patients were assessed by 3 experienced gastroenterologists. Lesions classified as P0 (no potential for bleeding) were not considered. The CIELAB color difference (ΔE) and visual analogue scales (VAS) were measured. The authors compared the sensitivity and detectability of ΔE and VAS of FICE1, FICE2 and blue mode for small intestinal lesions in 50 patients who underwent CE. FICE 1 and 2 had the highest sensitivity (100%) and specificity (97.3–100%) for vascular lesions. As for erosive/ ulcerative lesions, FICE 2 had the highest sensitivity (100%) and specificity (97.2%) for erosive/ulcerative lesions. WL had the highest sensitivity (90.9%) and specificity (87.1%) for tumors/polyps. FICE settings 1 and 2 showed significantly superior detectability of vascular lesions over WL. FICE setting 2 was significantly superior to WL in detecting erosive/ulcerative lesions. In the tumor images, there was no significant improvement with any of the settings compared with WL [11]. In another study, Dias de Castro et al. included 42 patients who underwent VCE for OGIB and negative SBCE examinations. The images were reviewed by four independent experienced gastroenterologists using standard white light examination. The findings were classified as P0, P1, and P2 lesions (non-pathological, intermediate bleeding potential, and high bleeding potential, respectively) and used as references. The patients were followed up for rebleeding and the images were re-reviewed. A review of the SBCE images using FICE 1 enabled the identification of previously unrecognized P2 lesions, mainly angioectasias in 21% of patients and P1 lesions, mainly erosions, in 62%. Among the patients who experienced rebleeding, 81% were diagnosed with P1 lesions with FICE 1 (*p* = 0.043), whereas 19% had confirmed nondiagnostic SBCE and only 6% had newly diagnosed P2 (plus P1) lesions. This study suggested that FICE 1 could increase the detectability of previously missed bleeding lesions compared to CE [12]. Imagawa et al. studied 50 patients by both conventional CE and FICE. The images were reviewed by two experienced endoscopists. Again, FICE1 and FICE2 endoscopy improved the detection of angioectasias at a statistically significant level (*p* = 0.0003, *p* < 0.0001, respectively). Detection of erosion, ulceration, and tumor did not differ statistically between conventional CE and CE-FICE [13]. Nogales Rincon et al. studied 41 VCE in 50 patients with small intestinal pathology classified in 3 groups of lesions (vascular lesions, erosions/ulcers and polyps/tumors). These lesions were evaluated by three independent, experienced reviewers using FICE compared with white light. FICE 1 mode significantly improved the visualization of angiodysplastic and vascular lesions in 88.9% of cases and that of erosions/ulcers in 77.8%. The FICE 2 mode improved detection of these lesions in 88.9% and 55.5% of cases, respectively. However, FICE 3 did not seem to provide any significant advantages. No significant improvement in the detection of polyps or tumors was noted [14]. Furthermore, in the study by Boal Carvalho et al., 60 patients with OGIB were included and SBCE studies were reviewed by five independent experienced gastroenterologists using FICE 1 and WL. The detection of small erosions and angioectasias (P2 lesions) was significantly higher with FICE 1 versus WL (*p* < 0.05). On the contrary, detection of ulcers and tumors was not significantly different between FICE and WL. The diagnostic yield of OGIB was significantly higher with FICE 1 (55% vs. 42%, *p* = 0.021), suggesting that virtual chromoendoscopy can increase the accuracy of the findings [5]. In another case series of 10 consecutive patients by Pohl et al., FICE1 achieved the best contrast between the vascular network and the background mucosa by enhancing the hypervascularity of small bowel mucosal lesions [15]. Konishi et al. studied a small cohort of 10 patients with OGIB. VCE images were reviewed by five independent gastroenterologists and the detection rates of small bowel lesions between FICE modes and WL were studied. The detection rates of vascular lesions, using FICE1 and FICE2 versus conventional CE, were significantly higher (*p* < 0.001). The detection of small erosions was also found to be significantly higher with FICE 1 and 2 and for red spots, it was more significant with FICE-1 (*p* < 0.001). FICE-3 did not seem to improve diagnostic accuracy versus the conventional imaging [16]. In a recent meta-analysis, FICE 1 was found to be helpful for angioectasias and ulcers/erosions and more specifically, in the delineation and the detection of lesions. FICE 1 was also useful to help identify excessive darkened bile in the ileum, which can be associated with abnormal bowel habit and diarrhea [17]. Kobayashi et al., in his study, had suggested that visualization of lesions was improved by FICE image analysis. Five physicians compared FICE images with the corresponding conventional images of 145 lesions obtained from 122 patients who underwent SBCE using all 3 FICE settings. With FICE1, visualization was improved in 83% of angioectasia images, 53% of erosion/ulceration images and 25% of tumor images. With FICE2, improvement was achieved for 87%, 25% and 20%, respectively. With FICE3, there was no significant improvement [18]. In a prospective study by Duque et al., 20 VCE were reviewed by 2 independent gastroenterologists using FICE 2 and CE. The FICE mode identified 17 additional erosions, (41.5%; *p* < 0.001), and 3 additional angiodysplasias (8.6%; *p* = 0.25). There was no significant difference in the detection of gross lesions between CE and FICE [19].

Regarding the blue filter (BF) mode, only a few retrospective studies were available with conflicting results. In 1 of them, 167 videos from 200 capsule endoscopies were reviewed by 2 experienced endoscopists. For all lesion categories, BF provided image improvement in 83% compared to white light, while with FICE 1, improvement was observed only in 34%, with a worse image observed in 55.9% of cases. FICE1 was effective in improving images of luminal blood. There was no significant image improvement in other lesion subgroups. With FICE 2, improvement was observed in 8.6%, but the image was worse in 77.5% and with FICE 3, improvement was observed in 7.7%, but the image was worse in 79.9%. The study concluded that BF offered better image enhancement in CE as compared with FICE [20]. However, Koulaouzidis et al., in a small cohort of 27 patients with IBD, reported that although the blue mode may enhance mucosal details, such as small mucosal breaks, it did not perform better than WL in the identification of the degree of small bowel inflammation using the Lewis score [21].

On the other hand, there are retrospective studies that have not shown any superiority of chromoendoscopy compared to conventional CE. In these studies, FICE was found to increase only visibility but not necessarily the detection rate of small bowel lesions. Kobayashi et al. studied a cohort of 24 patients who underwent VCE with a variety of small bowel abnormalities (tumors, angiectasias and ulcerative lesions). Three endoscopists reviewed the results in WL and each of the three different FICE modes and measured their sensitivity and specificity for small intestinal lesions. The overall sensitivity of CE was 94.4% with the standard mode, 90.7% with FICE1, 87.0% with FICE2 and 87.0% with FICE 3 and the overall specificity was 66.7%, 55.6%, 77.8% and 66.7%, respectively. No significant differences in the overall sensitivity were found. There was no significant difference between the standard and each FICE mode. In the per lesion analysis, FICE 1 had significantly increased the detection rate of angioectasias and ulcerative lesions compared to the standard mode (angioectasia, 25.7 vs. 21.0, *p* = 0.005; ulcerative lesions, 19.3 vs. 14.0, *p* = 0.06). However, FICE 1 had decreased the detectability of tumors compared to standard mode (4.3 vs. 10.0, *p* = 0.003) [18]. In another study by Gupta et al., in 60 patients who underwent SBCE for OGIB, no significant difference was found in the sensitivity or specificity of FICE for the detection of P2 lesions (lesions with high bleed potential) versus conventional imaging. FICE1 only improved the visibility of non-pathological P0 lesions (*p* < 0.01) [22]. Similarly, Matsumura et al. showed that the diagnostic yield for OGIB had not improved by FICE compared to conventional imaging. However, the total number of detected mucosal lesions was significantly higher using FICE (*p* < 0.01). The overall diagnostic yield in FICE sets 1, 2, 3 and conventional imaging were 51.9%, 40.7%, 51.9% and 48.1%, respectively, which showed no statistical difference [23]. Finally, a meta-analysis by Yung et al. on the clinical validity of FICE in SBCE concluded that the use of the three FICE modes did not significantly improve the delineation or detection rate of small bowel lesions overall. However, in pigmented lesions (angiectasias, ulcer/erosions), FICE1 performed better in lesion delineation and detection [6].

#### 3.1.2. Coeliac Disease

In a multicenter European study, FICE and blue filter were compared to WL for the detection of small bowel coeliac disease changes and the results were reviewed by five expert capsule reviewers. FICE and BF did not increase the sensitivity or specificity for the detection of coeliac changes. Sensitivity and specificity of conventional white light in the delineation of coeliac disease-related changes were 100%. FICE and blue light were not found to be superior to conventional white light in the delineation of macroscopic changes in coeliac disease [24].

### 3.2. Colon Capsule Endoscopy

One recent study by Nakazawa et al. assessed whether chromoendoscopy in colon capsule endoscopy (CCE) can assist in differentiating adenomatous and hyperplastic polyps non-invasively. The second generation CCE (CCE-2) has a high detection rate of approximately 84–94% for polyps ≥ 6 mm and 88–92% for polyps ≥10 mm, offering a pain-free, non-invasive examination. CCE-2 is equipped with flexible spectral imaging color enhancement (FICE) and blue mode (BM). Good differentiation was made possible between adenomatous and hyperplastic polyps by calculating the FICEΔE′ from the CCE images, with a sensitivity of 91.2% and specificity of 88.2%. A total of 52 lesions from 18 patients were assessed. The authors concluded that, if a lesion is <6 mm with FICEΔE′ ≤ 1.76 and the location is the rectum or sigmoid colon, it can be considered hyperplastic, and a watch-and-wait approach can be applied. If a lesion is 6–9 mm and the FICEΔE′ ≤ 1.76, colonoscopy may not be required immediately, as the lesion may be a hyperplastic polyp. However, the patient should undergo CCE or colonoscopy after one year or more [25].

## 4. Conclusions

In conclusion, there seems to be a paucity of studies that look at the role of virtual chromoendoscopy in VCE, especially considering the body of evidence on digital and conventional chromoendoscopy in fibreoptic luminal endoscopy. Most of the available studies have relatively small sample sizes and are usually single centered. Although several studies have shown that the visibility of pigmented lesions and especially angioectasias, erosions and ulcers can be enhanced with FICE 1 and 2 modes, in some studies, there was no evidence of significantly increased detection rates of those lesions, when images or capsule videos were reviewed by experienced capsule readers. FICE 3 and BF mode did not significantly increase the visibility of vascular lesions in most of the studies, although the data for the BF mode are particularly scarce (Table 1). The use of virtual chromoendoscopy for the differentiation of hyperplastic and adenomatous polyps in CCE-2 might be beneficial in the future and requires further investigation. Regarding coeliac disease, there was no additional benefit of virtual chromoendoscopy versus WLI [4]. There is still no strong evidence to support the routine use of chromoendoscopy in capsule interpretation, although in cases of subtle or indeterminate lesions, it may prove helpful for lesion characterization, delineation and detection. Further research should focus on whether chromoendoscopy can be used to assist inexperienced capsule endoscopists to visualize small vascular lesions in patients with OGIB, as these can often be easily missed. In addition, most studies have investigated the role of virtual chromoendoscopy in small bowel capsule endoscopy, and further studies are needed that focus also on other types of capsule endoscopy. In particular, the use of chromoendoscopy in colon capsule and polyp detection/characterization appears to be very promising. Similarly, its role in upper GI capsule endoscopy for lesion detection and assessment would be interesting, including evaluation of esophageal inflammation, Barrett’s esophagus and other upper GI lesions. In addition, its role has not been evaluated in the pan-intestinal (Crohn’s) capsule. Finally, the role of virtual chromoendoscopy as a training tool to assist trainees to detect subtle lesions and reduce the learning curve for capsule endoscopy training might be another area of interest for future research.

## Figures and Tables

**Table 1:** Summary of existing studies for capsule chromoendoscopy.

Authors	Year	Number of Patients Enrolled	FICE1	FICE2	FICE3	BF	Conclusions
Ogata et al. [4]	2018	24	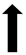	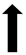	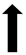	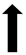	Increased detectability of erosions/angioectasias/small ulcers.
Da Silva et al. [2]	2018	22	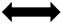	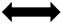	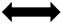	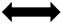	No significant difference was found in detectability of small bowel vascular lesions between WLI and CE.
Rimbas et al.	2015	250	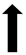	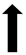	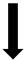	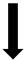	Increased detectability of ulceration with CE vs. WLI.
Nakamura et al. [3]	2012	50	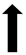	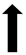	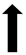	NA	FICE readings had statistically significant better sensitivity (91% vs. 80%) in angiodysplasia.
Sakai et al. [9]	2012	12	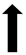	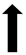	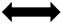	NA	FICE 1 and FICE 2 significantly improved the detectability of vascular lesions (angioectasias/erosions/ulcerations) (*p* < 0.01)
Cotter et al. [10]	2014	49	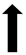	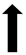	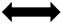	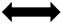	FICE 1 and FICE 2 significantly improved the delineation of vascular lesions.
Sato et al. [11]	2014	50/189 images	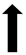	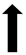	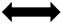	NA	FICE1 and FICE2 significantly improved the detectability of vascular lesions, with FICE 2 increasing the visibility of erosions/ulcers.
De Castro et al. [12]	2015	42	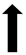	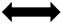	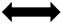	NA	FICE 1 increased the detectability of previously missed erosions/angioectasias vs standard mode CE (*p* < 0.05).
Imagawa et al. [13]	2011	50	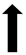	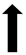	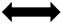	NA	FICE 1 and 2 increased the detectability of angioectasias only (*p* < 0.001).
Rinkon et al.	2013	41	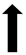	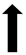	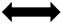	NA	FICE 1 and 2 significantly increased the detection of vascular lesions/erosions and ulcers.
Carvahlo et al.	2016	60	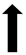	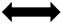	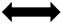	NA	FICE 1 significantly increased the detection of small erosions and angioectasias (P2 lesions)
Pohl et al. [15]	2010	20	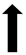	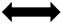	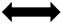	NA	FICE 1 increased the detectability of vascular lesions.
Konishi et al. [16]	2014	10	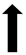	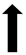	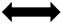	NA	Increased detectability of vascular lesions and especially erosions and angioectasias with FICE 1 and 2 (*p* < 0.001)
Aoyama et al. [17]	2020	134	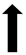	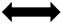	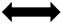	NA	FICE1 was found useful in detection of vascular lesions and especially angioectasias.Only study that FICE1 was proven helpful in diagnosis of bile acid associated diarrhea.
Van Gossum et al. [6]	2015	122				NA	Improvement in detection of vascular lesions with FICE 1 and 2.
Nakazawa et al. [25]	2021	51	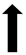	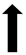	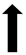	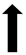	Improved differentiation of hyperplastic vs adenomatous polyps in colon capsule endoscopy.
Kalaouzidis et al.	2012	27	NA	NA	NA	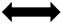	BF did not increase detectability of small bowel inflammation in IBD patients.
Krystallis et al. [20]	2011	200	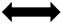	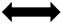	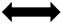	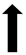	BF significantly improved visibility of luminal blood and vascular lesions.
Duque et al. [19]	2012	20	NA	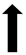	NA	NA	FICE2 mode identified significantly more erosions but no gross lesions(*p* < 0.01).
Kobayashi et al. [18]	2012	24	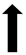	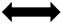	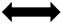	NA	FICE mode did not increase sensitivity or specificity over conventional CE for small bowel lesions overall.However, in per-lesion analysis, FICE 1 increased the detection of angioectasias (*p* < 0.05) but missed more tumors (*p* < 0.05).
Gupta et al. [22]	2011	60	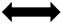	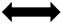	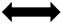	NA	No significant difference between FICE and WLI in OGIB.
Matsumura et al. [23]	2012	81	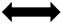	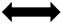	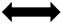	NA	No significant difference between FICE and WLI in OGIB.
Chetkuti et al.	2020	50	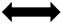	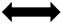	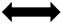	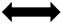	No significant difference between FICE and WLI in coeliac disease.

WLI: White light imaging, CE: chromoendoscopy, OGIB: obscure GI bleeding (↑: indicates significantly improved performance in lesion detection, ↓: indicates significantly lower performance in lesion detection, ↔: indicates no significant difference in lesion detection compared to conventional capsule endoscopy).

## Data Availability

Not applicable.
